# The execution rate of procedures to diagnose extrapulmonary tuberculosis in Botswana

**DOI:** 10.4102/phcfm.v12i1.2012

**Published:** 2020-01-27

**Authors:** Tantamika-Kabamba Mudiayi, Stephane Tshitenge, Botshelo T. Kgwaadira, Grace K. Nkubito

**Affiliations:** 1 Botswana National Tuberculosis Programme (BNTP), Ministry of Health and Wellness, Gaborone, Botswana; 2 Department of Family Medicine and Public Health, Faculty of Medicine, University of Botswana, Gaborone, Botswana

**Keywords:** extrapulmonary tuberculosis, execution rate of procedures, medical officers, Botswana

## Abstract

**Background:**

Extra-pulmonary tuberculosis (EPTB) accounts for about 20% of TB cases worldwide. Its diagnosis is challenging.

**Aim:**

This study meant to assess the prevalence of EPTB types, procedures to diagnose EPTB and medical officers’ (MOs) views on procedures performed in the diagnosis of EPTB over a 2-year period in Botswana.

**Setting:**

The study was conducted in 13 urban and rural facilities of 29 health districts in Botswana.

**Methods:**

This was a cross-sectional study that reviewed patients’ TB data and administered a questionnaire to MOs.

**Results:**

About 2 in 10 TB (*n* = 2996, 22.7%) cases were classified as EPTB. The most common site of EPTB was pleural (*n* = 1066, 36.7%) followed by lymph node (LN) (*n* = 546, 18.8%). A pleural tap was performed in 182 (17.0%) cases of pleural TB and a fine needle aspiration (FNA) in one-third (*n* = 160, 29.6%) of LN TB cases. There were statistical differences in work experience amongst MOs’ responses regarding their self-reported confidence to undertake basic procedures to diagnose EPTB such as pleural tap (*p* = 0.032) or FNA (*p* < 0.0001).

**Conclusion:**

This study reviewed and evaluated the proportion of EPTB and inquired about MO’s experience in managing EPTB. Despite MOs’ attendance at Botswana National Tuberculosis Programme (BNTP) TB case management (TBCM) training, the emphasis by the BNTP guidelines and availability of logistics, the execution rate of procedures to diagnose EPTB was still low in Botswana.

## Introduction

Africa accounts for 3 in 10 cases of tuberculosis (TB) worldwide and about 4 in10 cases of TB mortality globally.^1^ In 2014, Botswana registered 6542 cases with a death rate of 9% in a population of 2 million.^2,3^ Botswana has a high human immunodeficiency virus (HIV) prevalence in general population (18.5%; Botswana AIDS Impact Survey [BAIS IV] 2013). This significantly contributes to TB morbidity, as demonstrated by an high TB and HIV co-infection rate of 55% amongst TB cases in 2014.^2,4,5^ Extra-pulmonary TB (EPTB) accounts for about 20% of global TB cases, but disproportionately affects individuals with HIV in endemic areas and displays more diverse clinical manifestations and a worse outcome than pulmonary TB (PTB).^2,6,7,8^ It also presents more diagnostic challenges, especially in the developing world, because of scarcity of both diagnostic tools and procedures performed to obtain and analyse tissues.^4,9,10,11,12^ In Botswana, annual reports showed a steady rise in EPTB rates from 11% in 1990 and 13% in 2006 to 17% in 2008 and 19% in 2013 respectively.^2,11^

Whilst bacteriological confirmation constitutes an important indicator for adequate management of PTB and is well documented as demonstrated in annual performance reports, data on quality of diagnosis of EPTB are lacking.^2,4,12^

With partners’ support, Botswana National Tuberculosis Programme (BNTP) has invested in TB diagnostic processes, including a high laboratory coverage, the capacity-building of health care workers (HCWs) through training and clinical mentorship programmes and of late, the introduction of the Xpert MTB/RIF assay technology that has decreased the time to TB diagnosis and has improved diagnostic accuracy.^13^

The majority of medical officers (MOs) in Botswana are trained in an African setting. As TB is a common disease there, MOs are expected to be familiar with procedures to diagnose EPTB that include basic skills such as fine needle aspiration (FNA), lumbar puncture (LP), abdominal tap, pleural tap and excision biopsy of superficial tissue. Advanced skills to diagnose EPTB, such as pericardial tap, pleural biopsy, abdominal ultrasound and echocardiography, are generally performed by radiographers, radiologists or other specialists in referral hospitals.

This study aimed to auditing the quality of diagnosis of EPTB in Botswana. It meant to assess the prevalence of EPTB types, rates of procedures performed to diagnose it and MOs’ views on procedures performed in the diagnosis of EPTB.

## Methods

This was a cross-sectional study that reviewed patients’ TB data from 01 January 2010 to 31 December 2012 and administered a questionnaire to MO during the same period.

The BNTP supervises the TB programme. Botswana health services comprise 29 health districts that run TB preventive and curative activities. Laboratory and radiology services are available in all district hospitals, which have secondary care facilities, as well as primary hospitals. The BNTP’s role includes conducting TB training for trainers (TOT), TB case management (TBCM) training, clinical mentorship visits and monitoring and evaluation of the programme. The role of TOT is to implement in-service TB training at local level.

We selected 13 of the 29 health districts of Botswana. For this study, Gaborone and Francis town, the two major cities in Botswana, were selected. We also selected 11 districts, and amongst these, eight were rural and three were urban districts. The selected districts were Chobe, Ghanzi, Jwaneng, Kgalagadi North, Kgalagadi South, Kweneng East, Mabutsane, Ngamiland, Okavango, Tutume and Tonota. These districts had the highest TB notifications and accounted for about 60% of TB patients in Botswana.^2^

The BNTP guidelines (TBGs) provide guidance for diagnosis of EPTB. Extra-pulmonary tuberculosis was defined as the TB occurring outside the lung.^10^ Presumed diagnosis of EPTB was made when a patient presented symptoms and/or signs of TB related to an extra-pulmonary site, revealed by procedures (e.g. imaging or laboratory investigations) and followed by a decision taken by an MO to initiate anti-TB therapy. Diagnosis of EPTB is confirmed when the presence of *Mycobacterium tuberculosis* is demonstrated on specimens taken from affected anatomical sites by a positive culture, smear or Xpert MTB/RIF assay. A miliary TB was considered an EPTB if it signalled a disseminated TB. In Botswana, diagnosis of EPTB is the responsibility of MO. At a facility level, manual TB registers, electronic TB registers and patient TB cards help to capture routine patient information. Patient treatment cards are an important tool for assessing treatment adherence and keeping notes on disease evolution.

Patients’ data were collected manually by TB coordinators and TB focal persons after orientation for consistency by investigators using the ‘record review data abstraction form’ where relevant variables were recorded for easy and rapid data extraction. Information was collected from (1) unit (facility/district) TB registers, (2) patient TB treatment card and (3) Record Review Data Abstraction Form. Variables consisted of the year, district, health facility (HF), patients with TB, EPTB and their site.

Besides, TB coordinators and TB focal persons were assigned to disseminate information to MOs on the upcoming study at their sites; consent forms were then distributed. Self-administered questionnaires were issued to MOs willing to participate in the study; they were asked to return questionnaire in 2 weeks’ time in a designated box. Information collected was (1) demographic information (sex, work experience, TB experience, place of practice), (2) whether s/he was confident of TBCM, (3) when was last TB refresher course attended, (4) whether there was available logistics such as TBGs and medical equipment and (5) access to a supervisor or a mentor.

We summarised the data using mean value ± standard deviation (s.d.) for normally distributed variables, median ± interquartile range for skewed and frequency in percentage for binomial variables. Chi-square test was used to analyse association between MOs’ responses about their comfortability to perform basic and advanced skills and their work experience. R software, version 3.0.0, with R commander package, version 1.9-6, was used to capture and analyse the data. The level of statistical significance was *p* = 0.05.

Ethical clearance was obtained from the Health Research Development Council (HRDC) in the notification of IRB Review, and access to the records was obtained from the Ministry of Health & Wellness (MoH&W) in the letter of support for EPTB sent to respective District Health Management Teams (DHMTs).

### Ethical consideration

Ethical clearance was granted by Health Research Development Council (HRDC) in the notification of IRB Review Ref. No: PPME-13/18/1 Vol VIII (328), and access to records was granted by the Ministry of Health & Wellness (MoH&W) in a Letter of Support for EPTB Study – Ref: DPH 20/11 XII (22) sent to respective DHMTs.

## Results

The TB registers of 13 selected districts had a record of 13, 148 TB cases, of these 2996 (22.7%) cases were classified as EPTB. Amongst the EPTB cases, 1619 (54.0%) were males and 1377 (46.0%) were females.

Human immunodeficiency virus test was recorded in 2707 (90.4%) of EPTB cases, and TB/HIV co-infection was observed in 1920 (70.2%) of EPTB cases. Concomitant PTB/EPTB was found in 874 (29.1%) of EPTB cases, and of these cases, only 96 (11%) were confirmed bacteriologically. The EPTB case mortality rate was 10.5%.

**FIGURE 1:**
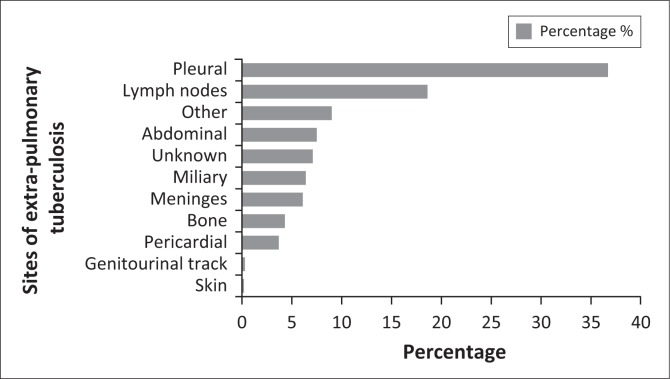
Frequency of extra-pulmonary tuberculosis, according to the site of infection, in 13 health districts of Botswana, *n* = 2996, from 01 January 2010 to 31 December 2012.

**FIGURE 2:**
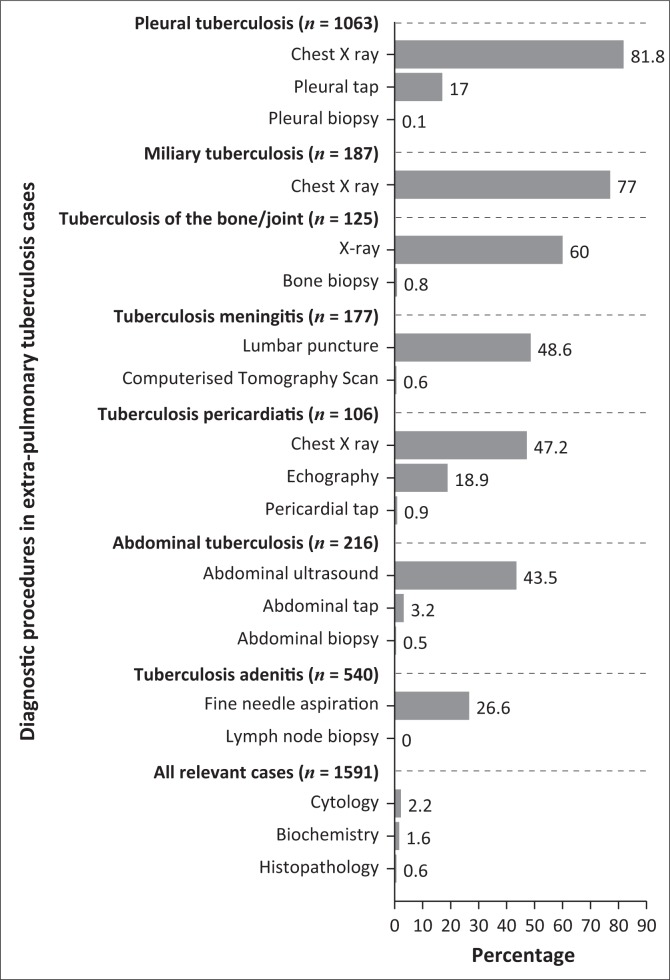
Performance of diagnostic procedures in extra-pulmonary tuberculosis cases, according to the site of infection in 13 health districts of Botswana, *n* = 2413, from 01 January 2010 to 31 December 2012.

The most common site of EPTB was pleural (*n* = 1066, 36.7%) followed by lymph node (LN) (*n* = 546, 18.8%) (Figure 1). Pleural TB was diagnosed using chest X-ray (CXR) in 870 (81.8%) of cases and a pleural tap was performed in 182 (17.0%) cases. To diagnose LN TB, an FNA was accomplished in one-third (*n* = 160, 29.6%) of the cases whereas close to half (*n* = 94, 43.5%) of abdominal TB was diagnosed using an abdominal ultrasound alone and/or in combination with abdominal tap (*n* = 7, 3.2%) and/or biopsy in one case (0.5%). In TB meningitis, an LP was performed in about half (*n* = 85, 48.6%) of the cases (Figure 2).

Of the 141 questionnaires distributed to MOs, 131 (93%) were returned. All the returned questionnaires were filled satisfactorily and included in the study. About three-quarters of MOs had either 6–10-year work experience (*n* = 48, 36.6%) or more than 10-year experience (*n* = 45, 34.4%) (Table 1). Seventy-nine (60.3%) MOs worked in a hospital setting whereas 50 (38.2%) MOs practised in primary health care (PHC) clinics, and two (1.5%) were from private practice HF.

**TABLE 1:** Sex distribution and work experience of medical doctors in 13 health districts of Botswana, from 01 January 2010 to 31 December 2012 (*n* = 131).

Work experience	Male	Female	Total	%
0–5 years	21	17	38	29.0
6–10 years	40	8	48	36.6
> 10 years	40	5	45	34.4

**Total**	**101**	**30**	**131**	**100**

About half (*n* = 63, 48.1%) of MOs responded that they had attended at least one BNTP’s TBCM training or a ToT course on TBCM (*n* = 61, 46.5%). Also, 6 in 10 (*n* = 83, 63%) MOs responded that they never participated in any kind of in-service TB training in their HFs, and 7 out of 10 (*n* = 85, 64.9%) MOs responded that they had the latest BNTP guidelines.

Also, two-thirds (*n* = 87, 66.4%) of MOs responded that they had previous TB management experience of at least 10 years. Amongst these, 31 (35.6%) responded that they managed TB cases every week, whilst 28 (32.2%) and 25 (28.7%) responded that they managed TB cases every month and on daily basis, respectively.

Whilst 105 (80%) MOs indicated that they felt ‘comfortable’ when managing PTB in general, 83 (69%) MOs felt ‘comfortable’ when also managing EPTB. The majority (*n* = 106, 81%) of MOs responded that they were ‘comfortable’ in the reading of abnormal CXR (Table 2). The majority (*n* = 105, 80.2%) of MOs felt ‘comfortable’ in conducting a pleural tap whereas more than half (*n* = 73, 55.7%) of MOs felt ‘comfortable’ in conducting an FNA. There were statistical differences in work experience amongst MOs’ responses regarding their self-reported confidence to undertake basic procedures to diagnose EPTB such as pleural tap (*p* = 0.032) or FNA (*p <* 0.0001).

**TABLE 2:** Extrapulmonary tuberculosis diagnostic skills and work experience of medical doctors in 13 health districts of Botswana, from 01 January 2010 to 31 December 2012 (*n* = 131).

Variable	Responses	Work experience						Total		Pearson’s chi-square *p*-value
		0–5 years		6–10 years		> 10 years				
		*n*	%	*n*	%	*n*	%	*n*	%	
**Basic EPTB diagnostic procedures**										
Abnormal chest X-ray	C	31	81.6	35	72.9	40	88.9	106	81.0	0.153
	NR	3	-	10	-	4	-	17	13.7	
	NC	4	-	3	-	1	-	8	5.3	
Spine X-ray	C	20	52.6	31	64.6	30	66.7	81	61.8	0.040*
	NR	8	-	14	-	12	-	34	24.9	
	NC	10	-	3	-	3	-	16	12.3	
Pleural tap	C	25	65.8	41	85.4	39	86.7	105	80.2	0.032*
	NR	6	-	6	-	4	-	16	12.2	
	NC	7	-	1	-	2	-	10	7.6	
LN FNA	C	14	36.8	31	64.6	28	62.2	73	55.7	< 0.0001*
	NR	2	-	11	-	11	-	24	18.7	
	NC	22	-	6	-	6	-	34	25.9	
Abdominal tap	C	28	73.7	45	93.8	41	91.1	114	87.0	0. 038*
	NR	3	-	2	-	2	-	7	5.3	
	NC	7	-	1	-	2	-	10	7.7	
**Advanced EPTB diagnostic procedures**										
Pericardial tap	C	5	13.2	3	6.3	11	24.4	19	14.5	0.002*
	NR	3	-	11	-	15	-	29	22.1	
	NC	30	-	34	-	19	-	83	64.4	
Pleural biopsy	C	5	13.2	4	8.3	11	24.4	20	15.3	0.006*
	NR	5	-	12	-	17	-	34	26.0	
	NC	28	-	32	-	17	-	77	58.7	
Total	*n*	38	-	48	-	45	-	131	100	-
	Percentage (%)	29.0	-	36.6	-	34.4	-	100	-	

*n*. number; EPTB, extra pulmonary tuberculosis; C, comfortable; NR, need a refresher course; NC, not comfortable; LN FNA, lymph node fine needle aspiration.

* *p* < 0.05 is statistically significant.

On the other hand, 2 in 10 MOs felt ‘comfortable’ in performing advanced skills to diagnose EPTB such as pericardial tap (*n* = 19, 14.5%) or pleural biopsy (*n* = 20, 15.3%). There was a statistical difference in work experience amongst MOs’ responses regarding their self-reported confidence to undertake advanced procedures to diagnose EPTB such as pericardial tap (*p* = 0.002) or pleural biopsy (*p* = 0.003).

## Discussion

This study audited the quality of diagnosis of EPTB in the 13 health districts of Botswana and evaluated the proportion and types of EPTB and diagnostic procedures. The study meant to describe the execution rate of procedures in diagnosing EPTB and assess the level of confidence of MOs to perform basic or advanced procedures in the diagnosis of EPTB.

In this study, 2–3 in 10 TB patients suffered from EPTB, whilst 3 in 10 EPTB patients had both PTB and EPTB. The proportion of EPTB varies from region to region, reflecting HIV prevalence. For instance, in our study, the majority of EPTB patients (70.2%) were HIV positive. Whilst countries such as Benin, with a low HIV prevalence (1%) compared to Botswana, reported an EPTB prevalence of 9%, and in Tanzania EPTB accounted for 15% in one study, and amongst them 58.3% were HIV positive.^12,13,14^

In this study, 36.6% EPTB cases were of pleural site and 18.8% of LNs site whereas both PTB and EPTB were observed in 29.1% of EPTB cases. African countries display similar patterns of EPTB sites of infection. As an illustration, a study from Tanzania reported a similar pleural TB prevalence of 44% and a higher LN TB prevalence of 31%.^14^ In a study from Cameroun, it was reported that concomitant TB and ETPB occurred in 20% of cases whereas a study conducted in Pakistan reported a dissimilar pattern, as LN and spine TB were the most diagnosed EPTB types in 60% of EPTB cases.^15,16^

In our study, 60.3% of MOs reported that they practised in a hospital setting and 38.2% reported that they practised in PHC clinics. A pleural tap was performed in about 2 in 10 (17.0%) TB pleural effusion cases, whereas an FNA was executed in only one-third (29.6%) of LN TB cases. Despite attendance to BNTP TBCM trainings (4 in 10 MOs), availability of BNTP guidelines (64.9% of cases) and MOs’ experience of at least 10 years (66.4% of MOs), a basic procedure such as pleural tap was performed only in 17% of pleural TB cases. Also, an LP was performed in only about half of TB meningitis cases. The low execution rate of such basic skills to diagnose EPTB appeared not to be generated because of lack of skills or logistics, as the majority of MOs felt ‘comfortable’ in performing a pleural tap (80.2%) or an FNA (55.7%). In Botswana, public health service is free of charge, and equipment to perform investigations are largely available. The low execution rate of LP could be explained by local cultural belief, as patients’ relatives often decline this procedure fearing death.

The low rate of procedures executed to diagnose EPTB also could be because of poor adherence of guidelines. Studies from various countries indicated inadequate adherence to institutions’ protocol on the procedures to diagnose EPTB. For instance, in a Tanzanian study, although available, essential tests such as histology or biochemistry were underutilised where only 18% of EPTB cases had laboratory-confirmed diagnosis. Also in Cameroun, the overall performance of investigations was 17%, whereas in a study from Pakistan, histology was carried out in 47%, radiology in 23% and microbiology in 9% of EPTB cases.^14,15,16^ Fear of delaying patient’s treatment initiation could explain the non-performance of procedures.

There is a need to review the support system of mentoring of MOs, continuous medical education (CME) and in-service lectures as currently implemented in the TB programmes which are mostly educational outreach visits (EOV). A review study reported that although more effective than educational materials or audit and feedback, EOV had modest effects on the effectiveness of guidelines’ implementation strategies, improving health professional practice and healthcare outcomes.^17^ Autonomy and self-direction by identifying educational needs and planning to meet individual learning objectives are important principles of adult learning. We recommend portfolio-based learning for MOs managing EPTB as it has proven to be a more effective method to learn skills in CME.^18^

In this study, there was a statistical difference in work experience amongst MOs’ responses regarding their self-reported confidence for undertaking basic procedures to diagnose EPTB such as pleural tap (*p* = 0.032) and FNA (*p <* 0.0001). Also, there was a statistical difference in work experience amongst MOs’ responses regarding their self-reported confidence for undertaking advanced procedures to diagnose EPTB such as pericardial tap (*p* = 0.002) and pleural biopsy (*p* = 0.003). Medical practice is grounded not only on scientific knowledge and training but also on clinical experience. With passage of time, MOs gain insights into professionalism and from transfer of skills.

Finding out *Mycobacterium tuberculosis* in pleural fluid or biopsy specimens remains the gold standard for diagnosing pleural TB. However, as MOs are not ‘comfortable’ to perform pleural biopsy and given the unavailability of biopsy kits in primary care, a lymphocytic predominant exudate and a high adenosine deaminase (ADA) level may be considered as diagnostics for diagnosis of pleural TB in a high TB prevalence setting such as Botswana. We recommend the availability of ADA analysis in PHC laboratories.^10,19^ Botswana TB guideline has recently recommended the use of Xpert MTB/RIF assay in the routine diagnosis of EPTB for a biopsy tissue sample, cerebrospinal fluid and urine, as it could improve the quality of EPTB diagnosis and reduce delay in initiation of treatment.^20^

Limitations of the study included those inherent to retrospective design such as incomplete patient records, potential for recall bias and inability to assess incidence. This study did not assess reasons for low rate of procedures’ execution to diagnose EPTB by MOs, or MO views on the CME and the support they had from the BNTP.

The study did not attempt to assess association between procedures’ performance rate and MOs’ confidence to perform these procedures. Further studies addressing these issues may be required.

## Conclusion

This study reviewed and evaluated the proportion and types of EPTB and inquired about MOs’ experience in managing EPTB. About 2 in 10 TB patients suffered from EPTB. Pleural and LN TB are the most commonly encountered types of EPTB. Despite MO attendance to BNTP TBCM training and the availability of TBGs and equipment, the execution rate of procedures to diagnose EPTB was still low in Botswana. We recommend portfolio-based learning for MOs managing EPTB, as it has proven to be an effective method to learn skills in CME.
